# The effect of sodium‐glucose co‐transporter 2 inhibitors on outcomes after cardiac resynchronization therapy

**DOI:** 10.1002/ehf2.14784

**Published:** 2024-04-22

**Authors:** Jonathan J.H. Bray, Marco Coronelli, Sam G.C. Scott, John A. Henry, Liam S. Couch, Mahmood Ahmad, Julian Ormerod, James Gamble, Timothy R. Betts, Andrew Lewis, Oliver J. Rider, Peregrine G. Green, Neil Herring

**Affiliations:** ^1^ Oxford Heart Centre Oxford University Hospitals, NHS Trust Oxford UK; ^2^ UCL Medical School University College London London UK; ^3^ Department of Cardiovascular Medicine University of Oxford Oxford UK; ^4^ Department of Physiology, Anatomy and Genetics University of Oxford Oxford UK

**Keywords:** Cardiac resynchronization therapy, CRT, Optimal medical therapy, Remodelling, SGLT2i, Sodium‐glucose co‐transporter 2 inhibitors

## Abstract

**Aims:**

The trials upon which recommendations for the use of cardiac resynchronization therapy (CRT) in heart failure used optimal medical therapy (OMT) before sodium‐glucose co‐transporter 2 inhibitors (SGLT2i). Moreover, the SGLT2i heart failure trials included only a small proportion of participants with CRT, and therefore, it remains uncertain whether SGLT2i should be considered part of OMT prior to CRT.

**Methods and results:**

We compared electrocardiogram (ECG) and echocardiographic responses to CRT as well as hospitalization and mortality rates in consecutive patients undergoing implantation at a large tertiary centre between January 2019 to June 2022 with and without SGLT2i treatment. Three hundred seventy‐four participants were included aged 74.0 ± 11.5 years (mean ± standard deviation), with a left ventricular ejection fraction (LVEF) of 31.8 ± 9.9% and QRS duration of 161 ± 29 ms. The majority had non‐ischaemic cardiomyopathy (58%) and were in NYHA Class II/III (83.6%). These characteristics were similar between patients with (*n* = 66) and without (*n* = 308) prior SGLT2i treatment. Both groups demonstrated similar evidence of response to CRT in terms of QRS duration shortening, and improvements in LVEF, left ventricular end‐diastolic inner‐dimension (LVIDd) and diastolic function (E/A and e/e′). While there was no difference in rates of hospitalization (for heart failure or overall), mortality was significantly lower in patients treated with SGLT2i compared with those who were not (6.5 vs. 16.6%, *P* = 0.049).

**Conclusions:**

We observed an improvement in mortality in patients undergoing CRT prescribed SGLT2i compared with those not prescribed SGLT2i, despite similar degrees of reverse remodelling. The authors recommend starting SGLT2i prior to CRT implantation, where it does not delay implantation.

## Introduction

Cardiac resynchronization therapy (CRT) is a well‐established treatment for heart failure with reduced ejection fraction (HFrEF) and wide QRS complexes. The European Society of Cardiology (ESC)^1^, ^2^, ^3^ and American Heart Association (AHA)^4^ guidelines recommend CRT in this cohort due to established benefits on hospitalization and all‐cause mortality.^5^ Major CRT trials were conducted in the early 2000s and were on optimal medical therapy of the time.^6^, ^7^ However, since these trials were performed, sodium‐glucose co‐transporter 2 inhibitors (SGLT2i) have become a key component of optimal medical therapy for HFrEF. SGLT2i have been demonstrated to reduce hospitalization and all‐cause mortality, although patients who had undergone CRT represented a small minority in the SGLT2i trials (<8% and 12% of participants in DAPA‐HF^8^ and EMPEROR‐reduced,^9^ respectively). There remains genuine uncertainty as to whether SGLT2i should be initiated before or after CRT implantation,^3^ and as a result, inconsistent clinical practice may be observed. Moreover, it is unlikely that randomized controlled trials for CRT will be re‐run using updated conventional medical therapy. We therefore performed a retrospective cohort study at a large tertiary centre to evaluate whether those patients undergoing CRT implantation obtained similar degrees of reverse remodelling and benefit in terms of hospitalization and mortality from an SGLT2i compared with those patients having CRT without SGLT2i.

## Methods

### Study design and participants

This retrospective, single‐centre cohort study was described *a priori* in the following protocol: https://rb.gy/6n7wn and conducted in accordance with STROBE reporting guidelines for cohort studies.^10^ Consecutive patients who underwent CRT implantation at the John Radcliffe Hospital, Oxford, UK, between 1 January 2019 and 1 June 2022, were entered into a procedural database. We chose 2019 as the year when SGLT2i were first demonstrated to have cardiorenal protective effects in randomized placebo‐controlled trials. CRT patients at our institution are enrolled on remote monitoring to assist follow up. Further information was extracted manually from electronic patient records (EPR) on the aetiology of participants' heart failure, information from ECGs on QRS duration and morphology, New York Heart Association class (NYHA) and co‐morbidity status including a diagnosis of hypertension, atrial fibrillation, diabetes mellitus, and chronic obstructive pulmonary disease (COPD). Information was collected on heart failure medications prescribed other than SGLT2i including angiotensin converting enzyme inhibitors (ACEi), angiotensin receptor blockers (ARB) alone and in combination with neprilysin inhibitors (ARNI), beta‐blockers, and mineralocorticoid receptor antagonists (MRA). Every author collecting data was a qualified medical doctor. All patients gave written informed consent to be included within the registry as a matter of routine when consent for the procedure was obtained, and the study was locally approved by our institutional board (clinical improvement module 8410).

### Intervention and comparison

It was recorded whether participants were prescribed oral SGLT2i medications prior to CRT implantation based on information from EPR. This information was extracted by authors from the NHS Spine, a nationwide clinical interface. The comparison was standard medical therapy without SGLT2i. During the period of rollout of SGLT2i, it took time to start the majority of patients with HFrEF on SGLT2i, and so patients not taking SGLT2i were not taking SGLT2i simply because they had not been reached yet by routine care.

### Outcomes

Measurement of electrophysiology included pre‐procedural QRS duration, pre‐procedural QRS morphology, post‐procedural QRS duration, and 6 month post‐procedural QRS duration. ECGs were considered if performed within 48 h before the procedure, 48 h after the procedure, and at follow up after the procedure around 6 months. Structural reverse remodelling in response to CRT was assessed by echocardiography before and 6 months after CRT including left ventricular ejection fraction (LVEF), left ventricular end‐diastolic inner‐dimension (LVIDd), and diastolic function (E/A ratio, e/e′ ratio). Outcomes also included hospitalization [for (a) heart failure and (b) all‐cause] and all‐cause mortality during follow up.

### Data collection and statistics

Data were collected into a pre‐formatted excel spreadsheet with appropriate security and pseudo identification. Continuous data were summarized with mean ± standard deviation, or median [interquartile range] if data failed a normality test (including visual assessment of residuals and the Shapiro–Wilks tests). Proportions were presented as total participants numbers with an event (*n*) and the associated percentage. Comparison of two independent groups were made using unpaired Student's *t*‐tests assuming unequal variance, or Mann–Whitney *U* tests where parametric assumptions are not met using IBM SPSS Statistics Version 27 (IBM Corp., Armonk, NY, USA). Variables with dichotomous data were compared using the chi‐square test. Event rates were explored using Kaplan–Meier survival curves and associated log‐rank (Mantel–Cox) tests for between factor overall comparison using SPSS. Graphical presentations were produced in Origin (Pro), 2021b (OriginLab Corp., Northampton, MA, USA).

## Results

**Figure 1:**
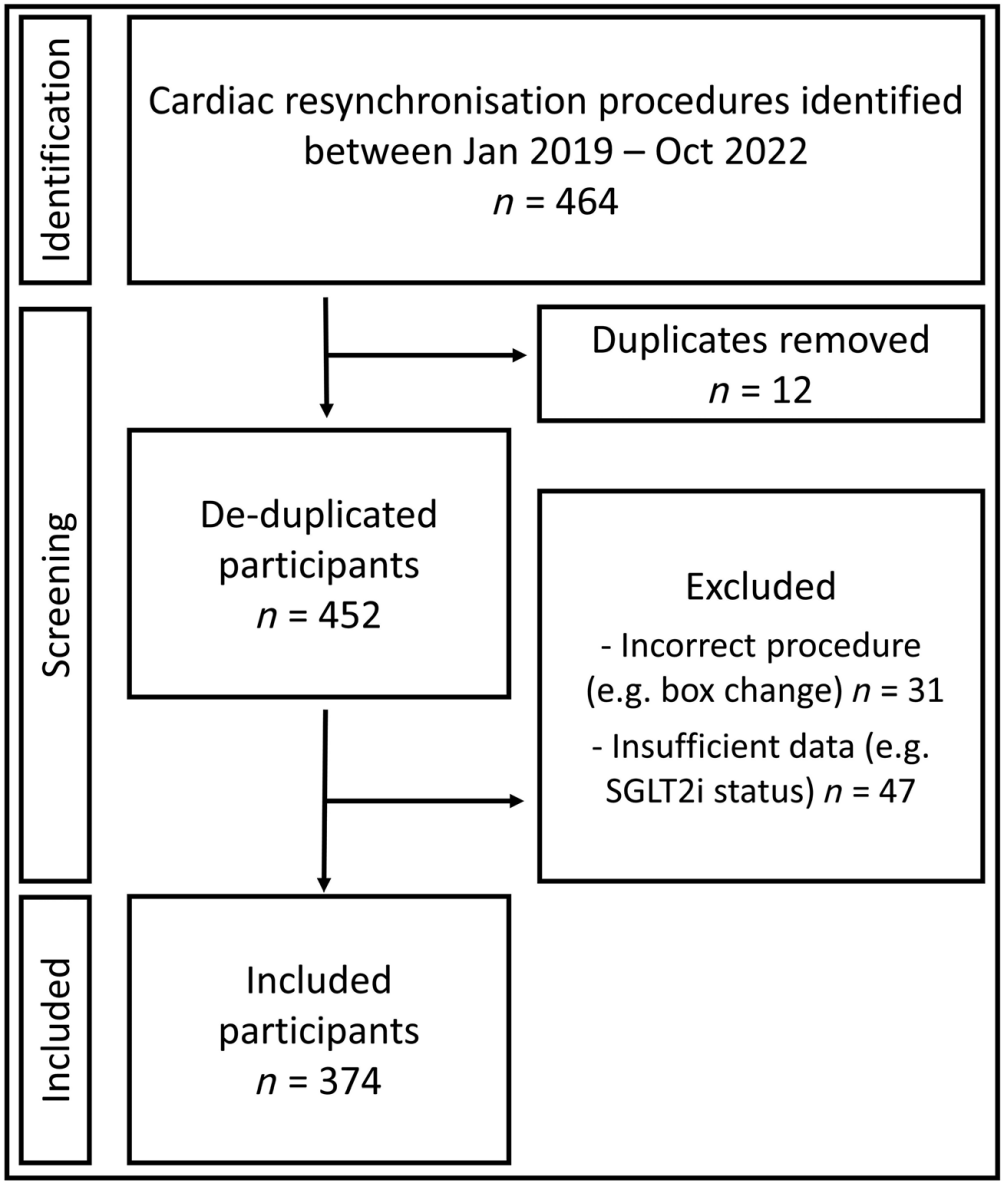
STROBE flow diagram of cohort creation and identification of eligible participants.

In total, 374 patients were included; *Figure* *1* shows a STROBE flow diagram for participants identified and excluded. Patients were aged 74.0 ± 11.5 years, 72.5% were male, with a LVEF of 31.8 ± 9.9%, QRS duration of 161 ± 29 ms and 57.8% with a left bundle branch block (LBBB). The majority had non‐ischaemic cardiomyopathy (58%) and were NYHA class II/III (83.6%). These characteristics were similar between patients with (*n* = 66) and without (*n* = 308) prior SGLT2i treatment. A significantly higher proportion of participants on SGLT2i had diabetes mellitus and atrial fibrillation as shown in *Table* *1*. The majority of participants underwent implantation of a CRT‐P (62.3%) versus CRT‐D, of these *n* = 32 received SGLT2i.

**Table 1:** Participant characteristics

Variable	SGLT2i (*n* = 66)	No SGLT2i (*n* = 308)	Total (*n* = 374)	*P* value
Age at procedure, years	72.3 ± 11.3	74.4 ± 11.5	74.0 ± 11.5	0.19
Sex, male (%)	50 (74.6)	230 (74.7)	280 (74.9)	0.89
LVEF, %	29.3 ± 8.9	32.3 ± 10.2	31.8 ± 9.9	0.06
Aetiology, *n* (%)				
Ischaemic	32 (48.4)	125 (40.6)	157 (42.0)	0.33
Non‐ischaemic	34 (51.6)	183 (59.4)	217 (58.0)	0.33
NYHA class				
I/II	17 (42.5)	56 (34.6)	73 (35.3)	0.22
II/III	38 (95.0)	135 (83.3)	173 (83.6)	0.06
III/IV	18 (45.0)	87 (53.7)	104 (50.2)	0.22
LBBB morphology, *n* (%)	40 (59.7)	176 (57.1)	216 (57.8)	0.63
QRS duration, ms	162 ± 26.9	160 ± 29.7	161 ± 29.2	0.07
Biventricular pacing, %	98.0 (95.8–99.0)	97.5 (92.1–99.0)	98.0 (92.7–99.0)	0.55
Heart failure medications				
ACEi/ ARB/ARNI	61 (92)	257 (84)	317 (86)	0.09
Beta‐blocker	52 (79)	237 (78)	288 (78)	0.88
MRA	44 (67)	173 (56)	224 (61)	0.09
Hypertension	29 (43.9)	126 (42.0)	155 (42.3)	0.77
AF	19 (28.8)	149 (50.0)	168 (46.2)	0.002
Diabetes mellitus	29 (43.9)	90 (29.9)	121 (33.0)	0.04
COPD	10 (15.2)	33 (11.1)	43 (11.8)	0.35
eGFR (mL/min/1.73 m^2^)	59.8 ± 21.1	58.1 ± 21.7	58.4 ± 21.6	0.55

ACEi/ARB/ARNI, angiotensin converting enzyme inhibitor OR angiotensin receptor blocker OR angiotensin receptor blocker with neprilysin inhibitor; AF, atrial fibrillation; COPD, Chronic obstructive pulmonary disease; eGFR, estimated glomerular filtration rate; LBBB, left bundle branch block; LVEF, left ventricular ejection fraction; MRA, mineralocorticoid receptor antagonist; NYHA New York Heart association symptomology class.

### Electrocardiogram response to cardiac resynchronization therapy

**Figure 2:**
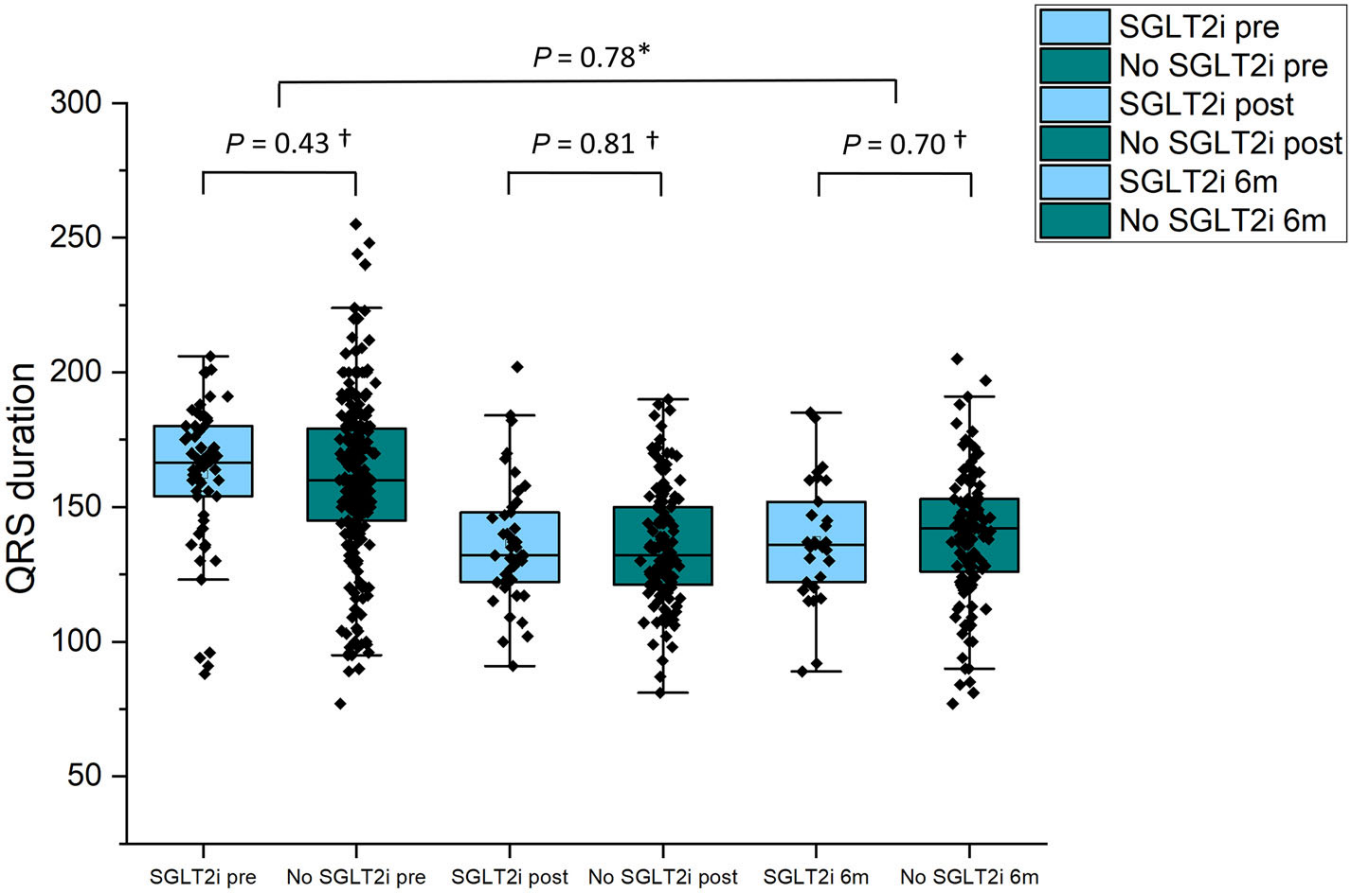
Box overlap graphs showing QRS duration before, after and 6 months after CRT implantation divided by participants prescribed SGLT2i and no SGLT2i. *Comparison of change in QRS before and after CRT implantation between SGLT2i versus no SGLT2i. ^†^Comparison of QRS between SGLT2i versus no SGLT2i.

QRS duration was significantly reduced following CRT implantation from 161 ± 29.2 (*n* = 317) to 135 ± 21.7 ms (*n* = 180, *P* < 0.001) and remained reduced at 139 ± 23.8 ms (*n* = 155, *P* < 0.001) 6 months after implantation. There was no difference in QRS duration between those taking and those not taking SGLT2i either before, immediately after, or at 6 months following CRT implantation as shown in *Figure* *2*. There was also no difference in the reduction in QRS duration from pre‐CRT to 6‐months post‐CRT implantation between groups (no SGLT2i and SGLT2i, respectively, 160 ± 29.7 and 162 ± 26.9 ms; *P* = 0.66 vs. 135 ± 21.7 and 136 ± 22.2 ms; *P* = 0.72).

### Echocardiographic response to cardiac resynchronization therapy

**Figure 3:**
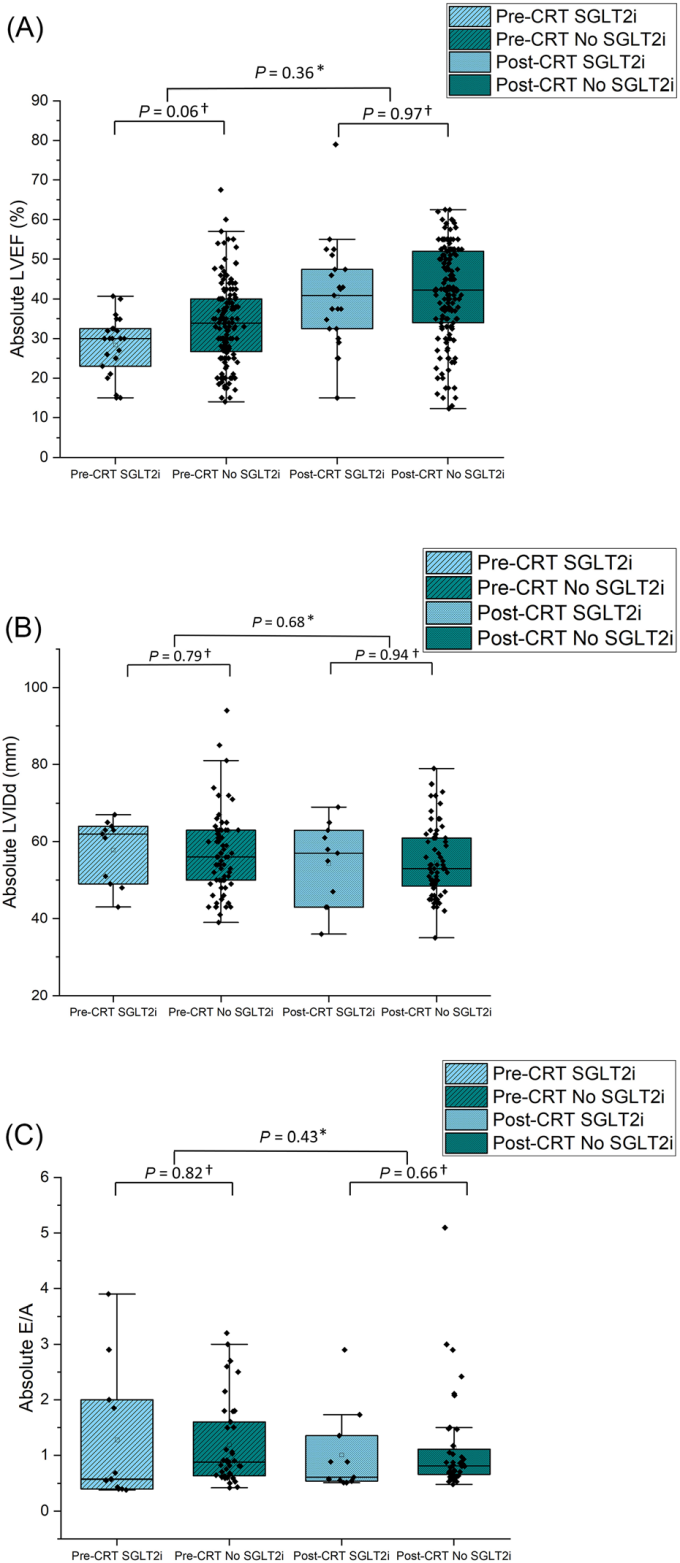
(A) Box overlap graphs showing absolute left ventricular ejection fraction (LVEF) determined by echocardiography before and after CRT implantation divided by participants prescribed SGLT2i and no SGLT2i. *Comparison of change in absolute LVEF before and after CRT implantation between SGLT2i versus no SGLT2i. ^†^Comparison of absolute LVEF between SGLT2i versus no SGLT2i. (B) Box overlap graphs showing absolute left ventricular internal diameter in end diastole (LVIDd) determined by echocardiography before and after CRT implantation divided by participants prescribed SGLT2i and no SGLT2i. *Comparison of change in absolute LVIDd before and after CRT implantation between SGLT2i versus no SGLT2i. ^†^Comparison of absolute LVIDd between SGLT2i versus no SGLT2i. (C) Box overlap graphs showing absolute E/A determined by echocardiography before and after CRT implantation divided by participants prescribed SGLT2i and no SGLT2i. *Comparison of change in absolute E/A before and after CRT implantation between SGLT2i versus no SGLT2i. ^†^Comparison of absolute E/A between SGLT2i versus no SGLT2i. (D) Box overlap graphs showing absolute e/e′ determined by echocardiography before and after CRT implantation divided by participants prescribed SGLT2i and no SGLT2i. *Comparison of change in absolute e/e′ before and after CRT implantation between SGLT2i versus no SGLT2i. ^†^Comparison of absolute e/e′ between SGLT2i versus no SGLT2i.

Following CRT implantation LVEF significantly improved from 31.9% ± 10.0% to 41.4% ± 12.2 (*P* < 0.001) (*Figure* *3* *A*). There was no significant difference between both groups in terms of the improvement in LVEF before and after CRT implantation (no SGLT2i and SGLT2i, respectively, 32.4 ± 10.2 vs. 29.7 ± 8.6%; *P* = 0.06 and 41.6 ± 12.2 vs. 40.6 ± 12.9%; *P* = 0.70).

LVIDd, E/A, and e/e′ were measured before and after CRT in 83 (22.2%), 51 (13.6%), and 57 (15.2%) participants, respectively. These metrics of remodelling and diastolic function did not change with CRT implantation, including LVIDd (57.0 ± 10.2 to 54.5 ± 9.2 mm), E/A 1.25 ± 0.85 to 1.23 ± 1.06), and e/e′ (13.5 ± 6.3 to 12.0 ± 5.4) (*Figure* *3* *B*–*D*). There was no significant difference between any of these metrics in SGLT2i versus control groups (SGLT2i: LVIDd 56.9 ± 10.5 to 54.5 ± 9.0 mm, E/A 1.31 ± 0.84 to 1.12 ± 0.77, e/e′ 13.4 ± 6.2 to 12.4 ± 6.5; no SGLT2i: LVIDd 57.3 ± 8.6 to 55.0 ± 10.4 mm, E/A 1.03 ± 0.88 to 1.64 ± 1.74, e/e′ 13.5 ± 6.4 to 12.0 ± 5.2).

### Hospitalization and mortality

**Figure 4:**
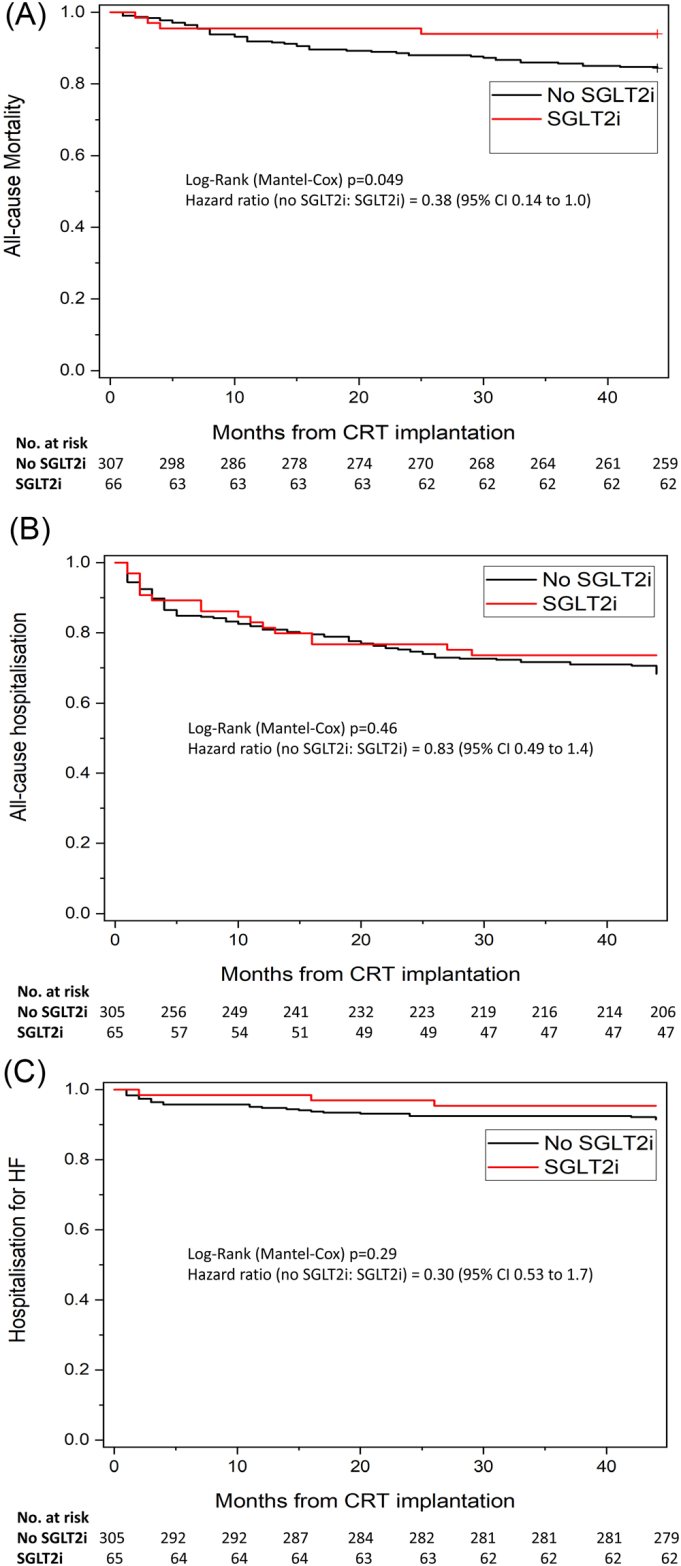
(A) Kaplan–Meier curve event rates for all‐cause mortality. (B) Kaplan–Meier curve event rates for all‐cause hospitalization. (C) Kaplan–Meier curve event rates for hospitalization for acute decompensation of heart failure.

Over a median follow up of 24.0 (13.1–34.8) months from CRT implantation, survival was 86.1%. However, those taking SGLT2i had significantly lower all‐cause mortality than those not taking SGLT2i (6.5 vs. 16.6%, HR 0.38 [95% CI 0.14 to 0.99], *P* = 0.049 (*Figure* *4* *A*). Hospitalizations occurred in 30.2% of participants for any‐cause and due to an acute decompensation of heart failure in 7.8% of participants (*Figure* *4* *B,C*). Neither outcome varied between SGLT2i and control [all‐cause: HR 0.83 (95% CI 0.49 to 1.4), *P* = 0.46; heart failure: HR 0.30 (95% CI 0.53 to 1.7), *P* = 0.29].

## Discussion

This study supports the hypothesis that patients prescribed SGLT2i who undergo CRT for heart failure benefit from similar electrical and structural responses to CRT but have a greater reduction in all‐cause mortality than patients undergoing CRT without SGLT2i. To the author's knowledge, this is the first study that has investigated this question directly. There was no difference in hospitalization from any cause or specifically related to acute heart failure following CRT implantation.

In the DAPA‐HF trial, only 7.5% of patients received CRT. In a post hoc analysis of both composite outcomes (HF hospitalization or cardiovascular mortality), it was unclear whether SGLT2 offered benefit in patients who had undergone CRT [hazard ratio (HR) 0.85, 95% CI 0.53 to 1.36 and HR 0.89, 95% CI 0.46 to 1.68, respectively].^11^ HF medication usage was similar in our cohort to SGLT2i trials and CRT trials (ranges – ACEi/ARB: 89% to 95%; BB: 66% to 96%; MRA: 54% to 72%).^6^, ^7^, ^8^, ^9^ Our data did not show any benefit of SGLT2i on markers of systolic and diastolic function with or without SGLT2i or CRT. There is evidence from meta‐analyses of randomized and non‐randomized studies suggesting that SGLT2i may improve LVEF, LVIDd, and e/e′.^12^, ^13^, ^14^ In randomized controlled trials SGLT2i improve rates of hospitalizations (all‐cause and for HF) and mortality (all‐cause and cardiovascular),^9^, ^11^ although in this study all‐cause mortality was reduced but hospitalizations were not. This may be secondary to different study sample sizes or differences in patient cohorts. The majority of the patients in our study were implanted with CRT‐P, and while there are no randomized controlled trials to date appropriately powered to compare CRT‐P and CRT‐D, it is possible that a CRT‐D population may have even lower mortality rates.^15^

From the data collected in this study, it is also possible to make an assessment as to whether there is an interaction between CRT implantation and SGLT2i on clinically meaningful outcomes.^15^, ^16^ CRT has been shown to exert cardiometabolic effects resulting in subsequent chronic reverse remodelling to improve cardiac function in those with heart failure.^1^, ^2^ It remains unclear how SGLT2i exert their cardiorenal protective effects and cardiac energetics have been proposed by some authors as a potential mechanism.^17^, ^18^ It may be hypothesized that should SGLT2i and CRT share a similar mechanism then an interaction between the two therapeutics may be seen. The recent EMPA‐VISION trial explored the role of SGLT2i in potentially altering cardiac energetics as a hypothetical mechanism by which SGLT2i can exert their cardiorenal protective effects.^19^ While the trial was small and potentially lacked the power to produce a definitive answer to this question, it demonstrated that empagliflozin did not improve cardiac energetics or change circulating serum metabolites associated with energy metabolism over 12 weeks, when compared with placebo in patients with HFrEF and heart failure with preserved ejection fraction (HFpEF). Our results suggest that patients undergoing CRT have the same improvements in systolic and diastolic function, and electrophysiology regardless of whether SGLT2i are prescribed or not.

Previous studies have also suggested that medical optimization of patients undergoing CRT is important, as in all other patients with HFrEF, and that this optimization is complimentary to the efficacy of CRT. Our data give further evidence that optimal use of full guideline directed medical therapy is of benefit in this cohort of people with heart failure.^2^ Nevertheless, CRT implantation should not be delayed to allow patients to be established on SGLT2i as the findings of this study do not show that SGLT2i contribute to additional reverse remodelling, and there are already barriers to referral for CRT implantation and delay is associated with progressively worse long‐term mortality.^20^, ^21^ Optimized implementation strategies have been proposed that includes improving identification and selection of eligible patients.

### Limitations

This cohort study is registry data, not randomized and therefore prone to the effects of confounding variables although it represents real world clinical practice at a large tertiary centre in the United Kingdom. We did not assess the subsequent adjustment (up or down titration) of HF medication following device implantation, as it is normal practice at our centre to perform CRT implantation after HF medication has been fully optimized. In terms of hospitalization and imaging follow up, there is also the potential for detection bias in patients moving out of the region and presenting to other hospitals, although it is unlikely that this bias would affect the intervention and control groups differently. Additionally, this potential bias would not affect mortality data, which is collected nationally. The proportion of patients with AF was incidentally noted to be higher in the non‐SGLT2i group, and there were insufficient data to perform adjustment. This could potentially have influenced outcomes although the percentage of biventricular pacing at follow up was similar in both groups suggesting a similar degree of resynchronization. The work is also limited by statistical power and the short follow up period and is intended to be hypothesis generating.

## Conclusions

Patients undergoing CRT prescribed SGLT2i appear to receive additional benefit in all‐cause mortality compared with patients not prescribed SGLT2i, despite similar response to CRT. We recommend starting SGLT2i prior to CRT implantation as part of optimal medical therapy, but SGLT2i initiation should not delay CRT implantation as our data suggest no evidence of additional reverse remodelling in this patient group.

## Conflict of interest

The authors do not declare any conflicts of interest.

## Funding

No funding was obtained to produce this study or manuscript. NH and OR are supported by British Heart Foundation Senior Clinical Research Fellowships (FS/SCRF/20/32005 and FS/SCRF/22/32014).
